# The effect of self-practicing systematic clinical observations in a multiplayer, immersive, interactive virtual reality application versus physical equipment: a randomized controlled trial

**DOI:** 10.1007/s10459-020-10019-6

**Published:** 2021-01-28

**Authors:** Helen Berg, Aslak Steinsbekk

**Affiliations:** Department of Public Health and Nursing, Norwegian University of Science and Technology, 7491 Trondheim, Norway

**Keywords:** Virtual reality, ABCDE approach, Group self-practice, Immersive, Interactive, Multiplayer

## Abstract

This study aimed to investigate whether group self-practice of systematic clinical observation using the airway, breathing, circulation, disability and exposure (ABCDE) approach in a multiplayer, immersive, interactive virtual reality (VR) application provided a non-inferior learning outcome compared to practicing with physical equipment in first-year medical and nursing students. The study was a non-inferior, parallel-group randomized controlled trial. After a 15-min introduction session on the ABCDE approach, all students were randomly allocated to practice ABCDE in groups of three for 20 min either in a fully immersive, interactive, multiplayer virtual reality application (the VR group) or with physical equipment (the TP group). The primary outcome was the number of students who documented all predefined observations in the correct order of the ABCDE approach on a practical test performed immediately after group practice. A total of 84% of all eligible students participated, with 146 students in the VR group and 143 in the TP group. On the primary outcome, 20% in the VR group and 21% in the TP group got everything correct (absolute difference 1% point, one-sided 95% confidence interval 1.0–8.8% points), showing non-inferiority of the virtual reality application. For other outcomes, the results were mostly similar between the groups. Group self-practice of the ABCDE approach in multiplayer, immersive, interactive virtual reality application was non-inferior to practice with physical equipment.

## Introduction

Group-based learning is frequently used in medical and healthcare education. This includes having students practice clinical skills in groups (O’Dunn-Orto et al. 2012; Tolsgaard et al. 2013), where they actively take part in the learning process (Kolb and Kolb 2005). The advantages of group practice are that students can learn from observing each other and help each other when they practice clinical skills, and it can be less resource intensive because it requires less facilitator or teacher time than does one-to-one instruction (Wulf et al. 2010; Räder et al. 2014).

One of the clinical skills that can be practiced in groups is systematic clinical observation, using the airway, breathing, circulation, disability and exposure (ABCDE) approach (Resuscitation Council UK 2015). A range of methods have been used to teach this approach (Thim et al. 2012; Smith and Bowden 2017), but we have not found any studies on the effect of using virtual reality (VR; Bremer et al. 2020). VR is “a computer generated digital environment that can be experienced and interacted with as if that environment was real” (Jerald 2015). With the use of a head-mounted display (HMD) described as an immersive technology, the user becomes completely occluded from reality and can experience a sense of presence in the virtual environment (Martirosov and Kopecek 2017). The user can interact with objects in the virtual environment using hand controllers.

To be able to practice as a group in VR, online, real-time, multiplayer features are necessary so that the group can be present and collaborate in the same environment (Liaw et al. 2018). Simulation-based activity is well suited for group learning (Hughes et al. 2016), and VR can potentially provide effective simulation-based group learning (McGrath et al. 2018; Kyaw et al. 2019). However, it is difficult to draw conclusions about the effect of different types of multiplayer VR applications because of methodological limitations in the studies (Liaw et al. 2018; Kyaw et al. 2019), and more research is, therefore, requested. VR has some disadvantages compared to real life when practicing clinical skills, and it can be expected that it would not give an effect above that of traditional practice with physical equipment, which has been widely used and developed over a long time. This is an argument for choosing a non-inferior design.

Therefore, the aim was to investigate if group self-practice skill training of the ABCDE approach in a multiplayer, immersive, interactive virtual reality application resulted in a non-inferior learning outcome in first-year medical and nursing students compared to using physical equipment.

## Method

### Study design

This was a non-inferior, parallel-group, open randomized controlled trial (RCT), which was conducted as part of a large trial also recruiting students to another RCT on the effect of individual practice simultaneously (Berg and Steinsbekk 2020).

The study was approved by the Norwegian Centre for Research Data (reference number 535088) and conducted from August to September 2019.

### Participants and recruitment

The inclusion criteria were first-year medical and nursing students who had started their study no later than 2 months before this study was conducted at one of the three campus sites of the Faculty of Medicine and Health Sciences, Norwegian University of Science and Technology.

The participants took part in a teaching programme integrated into their curriculum, and as part of this study, they were randomized in groups of three to take part in two different types of practicing the ABCDE approach. The group size of three was chosen based on research showing no difference in learning outcomes between groups of three, four or five (Rezmer et al. 2011), and when self-practicing, smaller groups would not be more resource demanding. The students were informed both verbally and in writing a week before and at the start of the teaching session, that they could consent to participate in the study at the end of the session. Those who attended the session were eligible, and those who consented were included.

### Randomization and allocation

Randomization had to consider the practical organization of the teaching, which included that batches of students had to be allocated to participate at separate times. To randomize these students, separate randomization lists were prepared for each batch using the Microsoft Excel RAND function. The lists were printed on identity stickers with identification (ID) numbers and codes for the type of practice in which the students were to participate. These were sealed in identical opaque plastic bags, which were mixed and randomly selected for each batch. The stickers were placed on the desk in ascending order according to the ID numbers. The allocation was done by asking each student entering the classroom to sequentially seat themselves at the desk with the lowest available ID number. They were not informed about what the allocation codes on their stickers meant.

When the introduction part was over, the participants were informed about where to go for their practice according to the allocation codes on their stickers by the person in charge of the session. The instructors for the self-practice part could not influence the allocation and were charged with ensuring that they got students with the right allocation.

### Interventions

The teaching session consisted of a 15-min introduction, 20-min group self-practice and approximately 15-min individual testing.

The main learning outcomes were to be able to keep to the order of the ABCDE approach, conduct the eight observations to be made (Table 1), and document the results of the observations. On the basis of the dialogue with those responsible for the curriculum at the study programmes and recommendations in guidelines and studies (Thim et al. 2012; Resuscitation Council UK 2015), it was decided which observations to include and the equipment (a digital blood pressure gauge, a digital oximeter, a digital ear thermometer, a clock, means for documentation and an overview of the ABCDE observations) to be used.

**Table1:** The information the students got regarding which eight observation to do and the order they should be done in

ABCDE algorithm	Observations
A—Airways	1: Observe if the airways are free. Document
B—Breathing	2: Count the respiration frequency (The number breaths per minute, one breath = inbreath + outbreath). Document
	3: Get the oxygen saturation using a digital oximeter. Document
C—Circulation	4: Get the blood pressure using a digital blood pressure gauge. Document
	5: Count pulse (the number of heart beats per minute). Document
D—Disability	6: Observe if the patient is conscious. Document
E—Exposure	7: Get the temperature using a digital ear thermometer. Document
	8: Observe if the skin is normal. Document

The introduction session, which was the same for all the participants, included a 6-min lecture on the ABCDE approach and a 7-min video made by the authors demonstrating how to do the ABCDE examination on an advanced simulator manikin (ABCDE introduction film; https://www.youtube.com/watch?v=8brQrQPg_2o).

The VR group received brief information on how to wear the VR equipment consisting of an head mounted display and hand controllers (Oculus Rift S or Oculus Quest; https://www.oculus.com/). The participants in the VR group were informed that they were supposed to practice as a group in the virtual patient room, with one student at a time performing the ABCDE procedure.

**Fig. 1:**
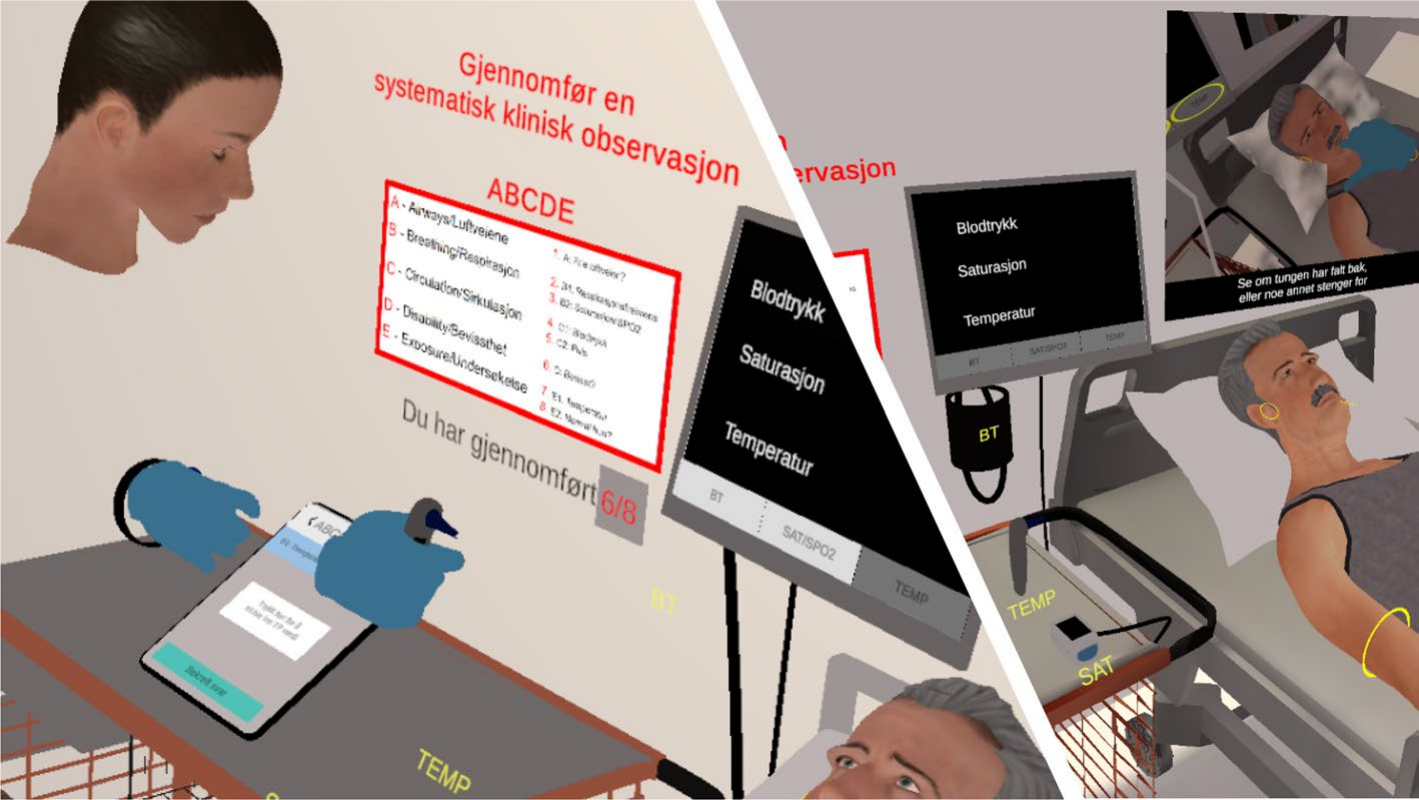
To the left, the look of the ‘head and hand’ avatar as seen by another player. To the right, the view for a player standing beside the patient

The authors, with hired help for programming in Unity (https://unity.com/) to make the application usable across platforms, made the ABCDE application (Table 2, video demonstrating the VR application; https://www.youtube.com/watch?v=5MbPHkcavmY). The application has a tutorial part on how to use the VR hand controllers and an ABCDE practicing part. In the tutorial part, the user is alone, and when the part is finished, the user is taken into the patient room where they meet the other group members. The first user entering the room is placed on the left side of the bed where all the equipment is lying and the observations are made (Fig. 1). The second user is placed on the right side of the bed and the third user at the foot-end. All the observations are done on a virtual patient lying on the bed, using virtual versions of the equipment. The two users not in reach of the equipment are activated through knowledge questions. The questions appear on digital boards in front of them each time the user on the left side of the bed starts a new observation. Instructions on how to make clinical observations are provided as a silent subtitled film on a screen on the wall. All the users can interact and communicate verbally and visually in real time and are present as avatars with head and hands only. When the user on the left side of the bed completes the ABCDE observations, feedback on performance is automatically generated and all the users automatically rotate one place to the right around the bed.

**Table 2:** Features in the practice part of the VR application (the VirSam ABCDE application)

VR-features	Explanation
Immersion	Be present in a 3D virtual room modelled from an equipped observation room and having 360-degree vision
Interaction	Virtual hands to pick up and move things and to get haptic response
Multi-player cooperation	Full interaction and audio and visual contact between the avatars of the group members. Avatar and user lip-synchronization
Rotation of positions	Users automatically rotated after each completion of an ABCDE examination to give all user the possibility to do clinical observations
Virtual patient (VP)	A healthy older male person lying on the bed half dressed, having visual response (eye blinking, head movement, open and close mouth, chest movement), haptic response (breath, pulse on the wrist), and changing clinical value responses to use of digital equipment (BP, temperature, O2 saturation). No vocal response
Haptic feedback	Vibration in the hand controllers when feeling the pulse (each heart beat) on the wrist, and when placing the hand on the chest (each respiratory intake)
Audio feedback	Inflation sounds from blood pressure gauge and “bip” from ear thermometer when the measure is ready (5 s)
Wristwatch	On left hand. Classic design showing real-time including seconds
Patient monitor	Monitor with touch screen buttons to get clinical values (BP, temperature, O2 saturation)
Documentation tablet	Tablet with touch screen buttons for responses, including numeric pad for entering clinical values and choice between predefined options
Instructions	A silent subtitle video running on a wall mounted screen showing how to do the observations, and a poster on the wall with the ABCDE observations
Feedback	When the user select that all documentations are done, a scoreboard appears with detailed feedback and a summary maximum of three stars, covering order of observations, whether all observations were done and if the values from the observations were correct

The participants allocated to group self-practice with physical equipment received a printed sheet with pictures of the equipment along with simple instructions on its technical use. They were also informed to practice as a group.

The minimum help from the instructors in both groups was to reflect a self-training situation.

### Data collection

The participants completed a baseline questionnaire when they entered the introduction session. The outcome data were collected through a questionnaire and a practical physical test, which was done individually after the group practice part.

After the practice, first, the participants individually answered questions about the correct order of the ABCDE approach, the eight observations, and their experiences with the different parts of the whole teaching session. There was no time limit, but the majority took approximately 6 min. Then, they individually performed a full physical ABCDE examination on an advanced simulator (the 3G or ALS Simulator; Laerdal Inc., Stavanger, Norway), with changing clinical values of a healthy person. On the bedside table, the same physical equipment as described above was available. They used a blank sheet of paper to document the observations, and this sheet was collected as the outcome measure for the practical test. The students were informed that they had 5 min to perform the ABCDE examination. One staff member was present, giving instructions about the time limit and equipment available to the students. They also showed where the values for blood pressure, O_2_ saturation and temperature were displayed on a monitor if the students did these observations. The staff helped students who struggled with technical issues. Otherwise, they did not interact with the students and were instructed to only answer “do as you think is best” if they asked anything. The staff were blinded to the allocation of students.

All data were scored independently by the first author (H. B.) and a third person hired for the purpose and then checked for accordance. Both were blinded to the allocation in this process.

### Implementation of the intervention

To monitor the implementation of the intervention, the technical problems encountered during self-practice were recorded. The participants were also asked how many times they completed the full ABCDE examination during the practice session (0, 1, 2, 3 or more).

### Outcome measures

The primary outcome was the number of participants who documented all eight observations in the correct order of the ABCDE approach on the practical test (yes/no).

Additional outcome measures were collected about the participants’ knowledge and performance of the ABCDE approach and their experience with the teaching session (see the “Results” section for details). The ten questions in the System Usability Scale (SUS) were transformed into one single score according to Brooke (1996) and were given a grade using the Curved Grading Scale (CGS; Lewis 2018).

### Statistics

Data for the sample size calculation came from previous studies testing clinical learning outcomes, indicating that a non-inferiority limit of 10 to 15% points is fair (Mpotos et al. 2014; Curran et al. 2015), and a limit of 13% points was decided. We conducted a pilot with 18 healthcare worker students in their second year at a vocational high school who had some experience in systematic clinical observation. Twenty percent of these students got everything correct on the primary outcome. We expected a similar outcome, arguing that the university students in our study had less practical experience but more experience in studying to master new tasks. With an expected outcome of 20% correct answers in both groups, with a non-inferiority limit of 13%, power (1-B) of 80% and significance level (alpha) of 0.05, 118 students were required in each arm, using the web calculator for non-inferior trials provided by Sealed Envelope (https://www.sealedenvelope.com/power/binary-noninferior/) (Julious 2004; Sealed Envelope Ltd. 2012).

Baseline variables are presented with descriptive statistics. As there were no deviations from the allocated groups and hardly any missing data, only one outcome analysis was performed using *t* tests for continuous variables (SPSS, v.26; IBM Corp., Armonk, NY, USA) and tests of proportions for categorical variables (StataMP, v.16; Stata Corp., Texas, USA). Results are presented as an absolute difference. For the primary outcome, a one-sided 95% confidence interval (CI) is reported according to the one-sided non-inferiority limit. The secondary outcomes are reported with a two-sided 95% CI.

## Results

### Recruitment and baseline characteristics

**Fig. 2:**
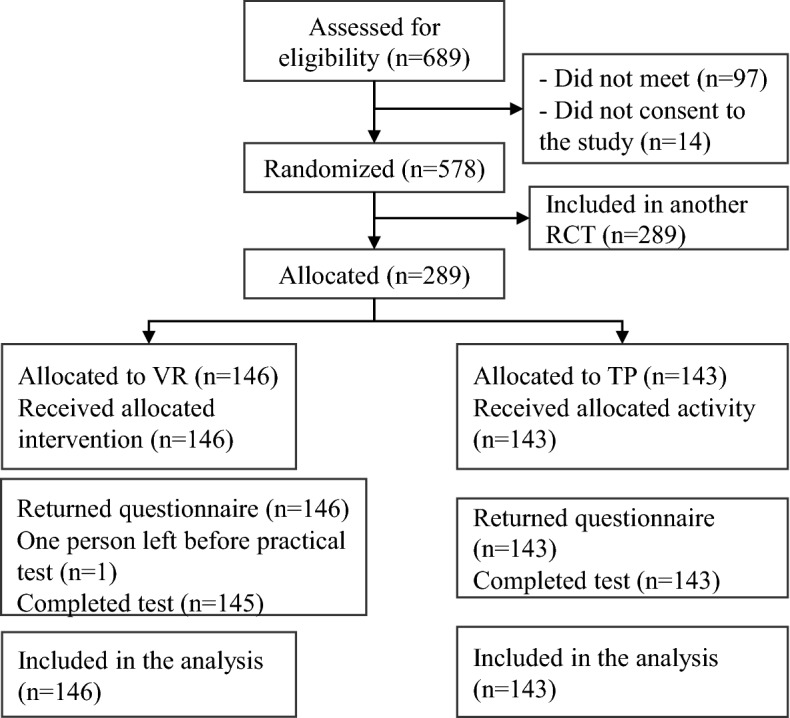
Flow of participants (*VR* virtual reality, *TP* traditional practice)

Of all 689 eligible first-year medical and nursing students in the large study, 289 participated in this study (Fig. 2). A total of 146 students were randomized to group self-practice in the VR application with virtual equipment (the VR group) and 143 to group self-practice with physical equipment (the TP group). The participants in both groups completed interventions and delivered outcome measures for analyses, except for one participant in the VR group who left before the practical physical test.

There were some differences between the groups at baseline (Table 3). The participants were younger in the TP group, with 39.7% younger than 20 years, compared to 19.4% in the VR group. More students in the TP group had experience with simulation, clinical observation and cardiopulmonary resuscitation.

**Table 3:** Baseline characteristics of the participants

Baseline variables	All, (N = 289)	VR group (N = 146)	TP group (N = 143)
Gender			
Male	43 (15.4)	16 (11.5)	27 (19.1)
Female	237 (84.6)	123 (88.5)	114 (80.9)
Age			
Under 20 year	83 (29.6)	27 (19.4)	56 (39.7)
20–24 year	166 (59.3)	90 (64.7)	76 (53.9)
Over 25 year	31 (11.1)	22 (15.8)	9 (6.4)
Study program			
Medicine	70 (24.2)	35 (24.0)	35 (24.5)
Nursing	219 (75.8)	111 (76.0)	108 (75.5)
Have you previously (number answering yes):			
Worked in health care	153 (54.6)	79 (56.8)	74 (52.5)
Been taught cardiopulmonary resuscitation (CPR)	230 (82.1)	107 (77.0)	123 (87.2)
Conducted systematic clinical observation	28 (10)	11 (7.9)	17 (12.1)
Been taught the ABCDE-approach	61 (21.8)	29 (20.9)	32 (22.7)
Used a blood pressure gauge	123 (43.9)	55 (39.6)	68 (48.2)
Counted respiration frequency on someone else	107 (38.2)	46 (33.1)	61 (43.3)
Tried virtual reality googles	88 (31.4)	38 (27.3)	50 (35.5)
Trained using a simulator manikin	112 (40)	52 (37.4)	60 (53.6)

The N for each variable can vary due to missing on some variables, N (%)

### Implementation of the intervention

There were no major technical or other types of practical problems in the implementation of the intervention. The only problem that was recorded included that the head mounted display had to be restarted two times because of lost tracking of the hand controller. A larger proportion in the TP group reported completing the full ABCDE examination more than two times during the practice session than did the VR group [25.2% in the TP group and 7.5% in the VR group; absolute difference 17.7% points (95% CI 9.3–25.9); data not shown].

### Primary outcome

**Fig. 3:**
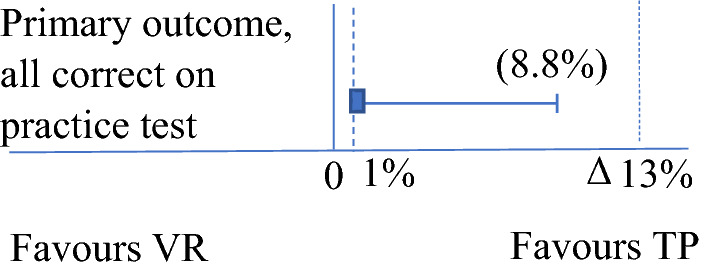
Primary outcome. 30 persons (21%) in the TP group and 29 persons (20%) in the VR group

The results of the primary outcome were that 29 (20%) participants in the VR group and 30 (21%) in the TP group got all eight observations documented in the correct order according to the ABCDE approach on the practical physical test. The absolute difference was 1% points, with the one-sided 95% CI upper level being 8.8%, thus demonstrating the non-inferiority of VR, as the confidence interval was within the non-inferior limit of 13% (Fig. 3).

### Secondary outcome

The outcomes on the two questions related to the correct order of the observations in the questionnaire were similar in the two groups [absolute difference 9.4% points (95% CI − 2.1 to 20.9) and 1.7% points (95% CI − 10.0 to 6.6)] (Table 4).

**Table 4:** Secondary outcomes measures concerning the ABCDE approach

Outcome measure	VR group N = 146 TP group N = 143	Observed values	Absolute diff. in % points (95% CI)	*P* value
Number of participants that in the questionnaire had				
All eight observations in correct ABCDE order	VR group	70 (47.9)	9.4 (− 2.1 to 20.9)	0.100
	TP group	82 (57.3)		
ABCDE in the right order	VR group	125 (85.6)	1.7 (− 10.0 to 6.6)	0.688
	TP group	120 (83.9)		
Number of participants in the practical test who				
Had all eight observations documented, but ABCDE in wrong order	VR group	7 (4.8)	0.1 (− 4.9 to 5.0)	0.977
	TP group	7 (4.9)		
Did not complete all eight observations	VR group	109 (75.2)	1.1 (− 11.1 to 9.0)	0.838
	TP group	106 (74.1)		
Did not complete all eight observations, but had the right ABCDE order on the documented observations	VR group	51 (35.2)	11.7 (0.4–23.0)	0.044
	TP group	67 (46.9)		
Wrote both the type of observation and the result of the observation	VR group	93 (64.1)	2.6 (− 13.8 to 8.6)	0.648
	TP group	88 (61.5)		
Number of participants in practical test with correct observation of (independent of order)				
Airways	VR group	144 (98.6)	0.7 (− 3.7 to 2.3)	0.635
	TP group	140 (97.9)		
Respiratory frequency	VR group	135 (92.5)	5.4 (0.6–10.3)	0.031
	TP group	140 (97.9)		
Saturation	VR group	137 (93.8)	2.7 (− 2.3 to 7.6)	0.291
	TP group	138 (96.5)		
Blood pressure	VR group	140 (95.9)	2.7 (− 1.0 to 6.5)	0.160
	TP group	141 (98.6)		
Pulse	VR group	117 (80.1)	4.5 (− 4.3 to 13.2)	0.318
	TP group	121 (84.6)		
Disability	VR group	87 (59.6)	6.4 (− 17.8 to 5.0)	0.270
	TP group	76 (53.1)		
Temperature	VR group	84 (57.5)	8.6 (− 20.0 to 2.9)	0.144
	TP group	70 (49.0)		
Skin	VR group	70 (47.9)	6.7 (− 18.1 to 4.7)	0.253
	TP group	59 (41.3)		
Average number of observations documented from practical test	VR group	Mean 6.3 SD (1.4)	Mean diff. 0.1 95 CI (− 0.162 to 0.392)	0.495
	TP group	Mean 6.2 SD (1.4)		
Average number of observations documented in the right order from A (Airways) in practical test	VR group	Mean 4.8 SD (2.4)	Mean diff. 0.4 95 CI (− 0.819 to 0.056)	0.151
	TP group	Mean 5.2 SD (2.1)		

Numbers are n (%) or mean (SD) with absolute difference between the groups and 95% confidence interval (95% CI)

The other outcomes on the participants’ knowledge and performance of the ABCDE approach were also similar in the two groups, except in the reporting of respiratory frequency where the students in the TP group performed better [97.9% correct in the TP group vs. 92.5% correct in the VR group; absolute difference 5.4% points (95% CI 0.6–10.3)] and in the number of students who did not complete all eight observations but had the correct order of the ABCDE approach on the documented observations, which also was in favour of the TP group [46.9% correct in the TP group vs. 35.2% correct in the VR group; absolute difference 11.7% points (95% CI 0.4–23.0)].

Concerning the participants’ experience with the teaching session, few thought they received enough training on the ABCDE approach before they started practicing, but the students in the VR group were more displeased [absolute difference 10.1% points (95% CI 0.8–19.4)] (Table 5). One in ten in the VR group thought they got enough time to practice compared to one in four in the TP group [absolute difference 15.3% points (95% CI 6.3–24.2)]. The TP group reported being more confident in conducting an ABCDE examination [absolute difference 8.8% points (95% CI − 0.7 to 18.2)]. The rest of the answers were similar between the two groups.

**Table 5:** Secondary outcomes measures concerning the student’s experiences with the teaching session

Outcomes measures	VR group N = 146 TP group N = 143	Observed values	Absolute diff. in %- points (95%CI)	*P* value
Number of participants who thought:				
They got enough training om the ABCDE before starting practicing	VR group	23 (15.8)	10.1 (0.8–19.4)	0.034
	TP group	37 (25.9)		
They learned what observations to do trough the video	VR group	72 (49.3)	8.4 (− 3.0 to 19.9)	0.151
	TP group	82 (57.7)		
They had enough time to practice	VR group	16 (11.1)	15.3 (6.3–24.2)	< 0.001
	TP group	37 (26.4)		
The way to practice was likable	VR group	100 (69)	7.4 (− 18.3 to 3.5)	0.186
	TP group	88 (62)		
The training and practice were a good way to learn the ABCDE approach	VR group	104 (71.2)	6.2 (− 17 to 4.5)	0.258
	TP group	93 (65)		
They were confident to conduct an ABCDE examination	VR group	25 (17.2)	8.8 (− 0.7 to 18.2)	0.070
	TP group	37 (26)		
System usability scale (SUS), range 0–100	VR group	Mean 72 SD (17.3)	Mean diff. 2.4 CI (− 6.1 to 1.4)	0.224
	TP group	Mean 74 SD (15)		

Numbers are n (%) or mean (SD) with difference between the groups and 95% confidence interval (95% CI)

The outcome on the SUS questionnaire was similar with a grade of C+ (SUS score 72) for the VR group, B− (SUS score 74) for the TP group on the Curved Grading Scale (Lewis 2018).

## Discussion

The participants in the TP group reported they had practiced the full ABCDE examination more than the VR group during the 20-min self-practice session. Nevertheless, self-practicing the ABCDE approach in groups in a multiplayer, immersive, interactive virtual reality application was non-inferior to self-practicing in groups with physical equipment. More students in the VR group reported that there was a lack of time to practice. Both groups thought that the way of practice was likable and that it was a good way to learn the ABCDE approach. The system usability test obtained a similar score in both groups, demonstrating the multiplayer, immersive, interactive virtual reality application to be of the same usability as using physical equipment for group practice.

The main strength of this study is its design. The study included the number of students required in the sample size calculation. The high proportion of eligible participants included makes the results generalizable to first-year medical and nursing students at comparable institutions. The groups had some differences at baseline; the TP group had younger students, who reported more experience with the use of simulation and VR which was considered to be a disadvantage for the VR group. There was no blinding of the students due to the nature of the study, but a recent meta-epidemiological study found that blinding did not influence the outcome (Moustgaard et al. 2020). It is also a limitation that the study tested only one type of VR application, with no follow-up for knowledge retention.

The practical test was physical and performed with the same physical equipment used by the TP group, and the students in this group reported having practiced the full ABCDE examination more. Still, it was found that practicing in VR gave a non-inferior learning outcome compared to practicing with physical equipment. Furthermore, emphasis was put on making an intervention that was adaptable and sustainable for use in ordinary teaching settings. VR solutions are scalable as the equipment needed have limited costs and the programmed VR application has no upper limit in numbers of users. This provides initial evidence for the efficiency of self-practicing procedure skills in a multiplayer, immersive, interactive virtual reality application.

We have not found other studies investigating this type of VR application. Studies on group practice in VR that use desktop solutions, have also found desktop VR solutions to give similar outcomes as traditional training (Youngblood et al. 2008; Khanal et al. 2014; Liaw et al. 2018). Together with our study, this lends support to the claim that VR can be used successfully for group training in skills typically trained involving the participants themselves or simulator manikins. This is further supported by recent studies, reviews and meta-analyses, which have demonstrated that single-player VR applications also provide similar or better outcomes than do the control intervention (Bracq et al. 2019; Kyaw et al. 2019; Berg and Steinsbekk 2020).

There are probably several reasons the VR group got a non-inferior learning outcome despite less practice. Studies have shown that students practicing in a group learn from observing each other and helping each other when they practice clinical skills (Wulf et al. 2010), and the VR application provided this by multiplayer functions, allowing the students to see and interact with each other. As becoming passive can make students lose focus, the VR application provided the users in the observer positions with knowledge questions about the ABCDE approach, which is in line with the findings of other studies showing that activity for all users is important in multiplayer VR (Creutzfeldt et al. 2016).

Most importantly, perhaps, the VR application also gave feedback to the users. Feedback is important for the learning outcomes of clinical skills (Khanal et al. 2014; Sahu et al. 2019). Feedback was provided to both the users answering the knowledge questions in passive positions and more extensively the users doing clinical observations. Although the participants could provide feedback to each other in both groups, only the VR group received autonomous feedback. This study does not have data on the effect of feedback in itself, but the importance of feedback in general points to the need for studies on the effect of VR with and without autonomous feedback.

There are studies discussing the importance of avatar realism in multiplayer, immersive, interactive environments, which argue that simple geometric avatars can support successful collaboration on physical tasks between users (Steed and Schroeder 2015; Roth et al. 2016). Other research has found that the looks of avatars affect how users respond to each other (Waltemate et al. 2018). The avatars in our application are gender neutral and have head and hands only, but they have lip synchronization, so the other user could see who was taking.

The strength of the ABCDE VR application developed for this study is the combination of multiplayer possibilities, immersion through the head mounted display and interaction with the environment using hand controllers, all of which contribute to making the experience relevant. This is different for most VR applications used for clinical practice, which is either immersive or interactive (Liaw et al. 2018; Bracq et al. 2019; Kyaw et al. 2019), and the immersive VR applications lacking the multiplayer dimension. Because of being developed in 2019, the application used in this study benefits from the rapid development of technology and the availability of VR solutions for the consumer market. As such, the application is in the forefront, but given the speed of innovations in this field, new and improved possibilities are likely to be available for further improvement of VR solutions to practice clinical skills.

## Conclusion

To the best of our knowledge, this is the first study on the effect of multiplayer, immersive, interactive virtual reality application for self-directed group-based procedure practice. With a time limit of 20 min for practice, group practice in VR gives each individual student less opportunity to practice the ABCDE approach compared to using physical equipment probably because of unfamiliarity with doing observations in VR. Still, group self-practice of the ABCDE approach in a multiplayer, immersive, interactive virtual reality application was non-inferior to practice with physical equipment.
